# Preparation and Thermal Properties of Propyl Palmitate-Based Phase Change Composites with Enhanced Thermal Conductivity for Thermal Energy Storage

**DOI:** 10.3390/polym15153192

**Published:** 2023-07-27

**Authors:** Linzhi Yin, Min Zhao, Rui Yang

**Affiliations:** Department of Chemical Engineering, Tsinghua University, Beijing 100084, China; yinlinzhibox@163.com (L.Y.); yangr@mail.tsinghua.edu.cn (R.Y.)

**Keywords:** propyl palmitate, phase change composites, expanded graphite, thermal conductivity, thermal properties, latent heat

## Abstract

Phase change materials (PCMs), which can absorb and release large amounts of latent heat during phase change, have been extensively studied for heat storage and thermal management. However, technical bottlenecks regarding low thermal conductivity and leakage have hindered practical applications of PCMs. In this paper, a simple, economical, and scalable absorption polymerization technique is proposed to prepare the polymethyl methacrylate/propyl palmitate/expanded graphite (MPCM/EG) phase change composites by constructing the microencapsulated phase change materials (polymethyl methacrylate/propyl palmitate, MPCM) with core-shell structures in the three-dimensional (3D) EG networks, taking propyl palmitate as the PCM core, polymethyl methacrylate (PMMA) as the shell, and long-chain “worm-like” EG as the thermally conductive networks. This technique proved to be a more appropriate combinatorial pathway than direct absorption of MPCM via EG. The MPCM/EG composites with high thermal conductivity, high enthalpy, excellent thermal stability, low leakage, and good thermal cycle reliability were prepared. The results showed that the MPCM-80/EG-10 composite demonstrated a high thermal conductivity of 3.38 W/(m·K), a phase change enthalpy up to 152.0 J/g, an encapsulation ratio of 90.3%, outstanding thermal stability performance, and long-term thermal cycle reliability when the EG loading is 10% and propyl palmitate is 80%. This research offers an easy and efficient approach for designing and fabricating phase change composites with promising applications in diverse energy-saving fields, such as renewable energy collection, building energy conservation, and microelectronic devices thermal protection.

## 1. Introduction

Energy is an important guarantee for industrial production and transportation. The use of fossil energy has fueled a century of rapid global development. However, the energy conversion process generates large amounts of waste; therefore, the development and storage of clean and sustainable energy resources have become increasingly urgent [1]. Among thermal energy storage (TES) systems, phase change materials (PCMs) are the most attractive materials for improving energy utilization efficiency owing to their high thermal storage capacity, ease of operation, and transformative industrial potential [2,3,4]. PCMs have been extensively studied in recent years in the fields of solar energy harvesting, building energy efficiency, smart textiles, waste heat recovery, electronic components, and food and drug industries [5,6,7,8,9,10]. PCMs can be divided into two categories, inorganic and organic PCMs, based on their chemical composition. Compared with inorganic PCMs, organic PCMs have the advantages of high energy storage capacity, flexible phase change temperature, good chemical stability, low supercooling, non-corrosiveness, and reasonable cost [11,12,13]. However, the low thermal conductivity, volatility, and leakage problems of typical organic solid–liquid PCMs with small molecular weights severely limit their practical applications [14,15,16]. Therefore, it is highly crucial to improve the thermal conductivity and develop appropriate encapsulation structures for organic PCMs.

To address the leakage problem, the encapsulation of PCMs into shell materials [17,18,19], porous supporting materials [20,21,22], and polymer matrices [23,24,25] has been considered as a universal strategy. In particular, microencapsulated PCMs with a typical core-shell structure, which increases the heat transfer area, controls volume changes, provides enhanced thermal stability, and prevents leakage of PCMs during heat uptake/release cycles, is the most commonly used method to fabricate advanced PCM composites [26,27,28]. Commonly used shell materials are mainly polymers or inorganic materials. In this regard, poly(methyl methacrylate) (PMMA) and acrylate copolymers [29,30], polyurethane (PU) [31], polystyrene (PS) [32], urea-formaldehyde resin [33], CaCO_3_ [34], SiO_2_ [35], TiO_2_ [36], and magnetic Fe_3_O_4_ [37] are the most commonly used shell materials. However, the low thermal conductivity of polymeric shells leads to difficulties in heat transfer between PCMs and circumstances in application scenarios where high-speed heat transfer is required. Although inorganic shell materials can improve the thermal conductivity of PCM microcapsules to some extent, the improvement is still limited [38]. In addition, inorganic shell materials are not easily deformed and are difficult to adapt to the volume changes of PCMs during the phase change process, making them susceptible to breakage and causing leakage.

To improve the thermal conductivity, the introduction of highly thermally conductive carbon-based materials, such as expanded graphite (EG) [39,40,41], graphite/graphene nanosheets [42,43,44], graphene oxide [45,46], and carbon nanotubes [47,48], into PCMs to form effective heat transfer channels in the composites is the most convenient and commonly used method. In addition, fillers, such as boron nitride [49], metal nanoparticles [50], etc., are also included. Among various potential fillers, EG is the most attractive and effective filler for its typical three-dimensional (3D) porous structure and large specific surface area, high thermal conductivity, and powerful absorption capacity for organic PCMs, which can be used as a container for PCMs to improve the thermal storage performance and impart high thermal conductivity, good encapsulation, and shape stability to PCM composites [51,52,53,54]. However, due to the presence of desorption, the absorption by capillary forces through EG is not sufficient to hold the PCM core, especially after thermal cycles. Therefore, additional encapsulation is still required to prevent leakage. In addition, the enhanced thermal conductivity conferred by thermally conductive fillers demands large amounts of fillers to form a thermally conductivity network [55]. The increase in thermal conductivity comes at the cost of a decrease in thermal storage capacity [56]. Obtaining high thermal conductivity while maintaining a high phase change enthalpy seems to be in conflict with the commonly used preparation methods. In conclusion, the preparation of PCM composites with a comprehensive balance of properties, i.e., simultaneously high thermal conductivity, high enthalpy, and leakproofness, remains a major challenge.

In this paper, we propose an absorption polymerization technique, which combines the advantages of EG as the thermally conductive porous support material and PMMA-encapsulated propyl palmitate (PCM core) to form core-shell-structured microcapsules with polymer shells in the EG networks, and thus, the PMMA/propyl palmitate/EG (MPCM/EG) phase change composite has been successfully prepared. Propyl palmitate (a fatty acid ester) is used as the PCM because it is derived from renewable resources, which are abundant, low cost, and environmentally friendly. This technique preserves the complete “worm-like” 3D network structure of EG, and microcapsules with a core-shell structure are formed in the EG network, thereby achieving high thermal conductivity, high enthalpy, excellent thermal stability, and low leakage of PCM composites at the same time. The produced MPCM/EG composites have excellent comprehensive properties: thermal conductivity of 3.38 W/(m·K), latent heat up to 152.0 J/g (encapsulation rate of 90.3%), outstanding thermal stability, and long-term thermal cycling reliability. The development of absorption–polymerization technology opens up a new way of manufacturing high-performance PCM composites, which have broad application prospects in the fields of thermal energy storage/release, intelligent buildings, and waste heat recovery management.

## 2. Materials and Methods

### 2.1. Materials

Propyl palmitate (industrial grade, with an average phase transition temperature of 18.3 °C and an average latent heat of 168.3 J/g) was chosen as the PCM core material. Methyl methacrylate (MMA, analytical purity, BASF New Materials Ltd., Shanghai, China) was used as the monomer and washed with NaOH solution to remove the inhibitor before polymerization. Expandable graphite was supplied by Beijing Warwick Chemical Co., Ltd., Beijing, China. Ammonium persulfate (APS, Macklin Biochemical Co., Shanghai, China) and sodium hydrogen sulfite (NaHSO_3_, Meryer Chemical Technology Co., Ltd., Shanghai, China) were chosen as redox initiators. Span 80 and Tween 80 were provided by Modern Oriental Technology Development Co. (Beijing, China) and were chosen as surfactants.

### 2.2. Preparation of Phase Change Composites

#### 2.2.1. Preparation of Expanded Graphite (EG)

EG is obtained by thermal exfoliation of expandable graphite. A muffle furnace was preheated to 900 °C, and then, a certain amount of expandable graphite was heated in the muffle furnace for 2 min to obtain the “worm-like” porous EG material.

#### 2.2.2. Preparation of PMMA/Propyl Palmitate Microcapsule (MPCM)

**Scheme 1:**
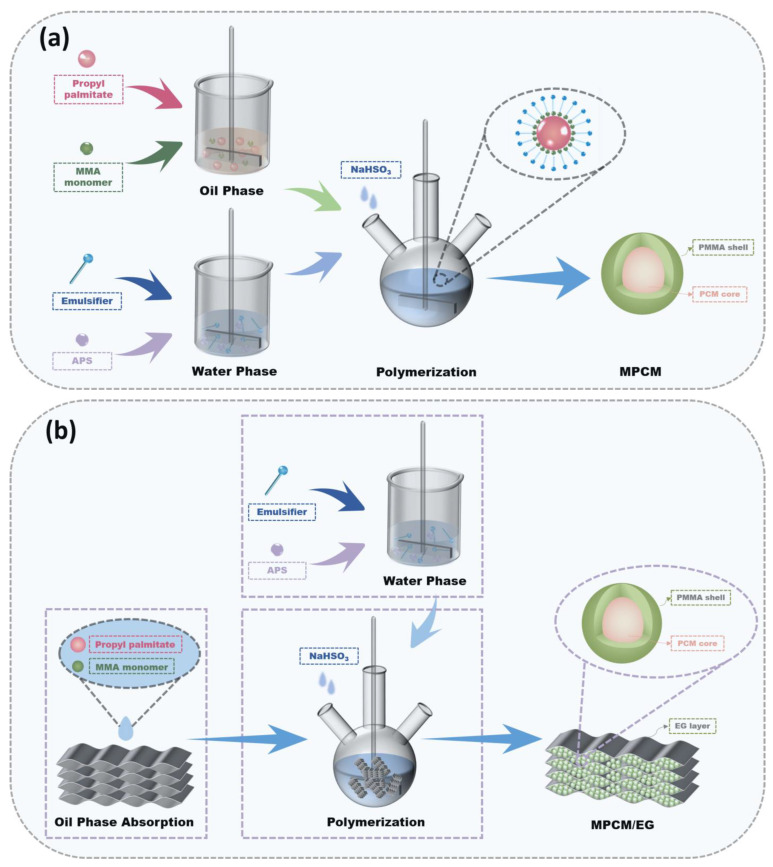
(**a**) Schematic illustration of the synthesis of PMMA-microencapsulated phase change materials (MPCMs). (**b**) Schematic illustration of the synthesis of MPCM/EG phase change composites.

The preparation of PMMA-microencapsulated phase change materials (MPCMs) by redox-initiated polymerization is shown in Scheme 1a. First, propyl palmitate and MMA monomer were mixed to form the oil phase; 1.5 g of emulsifier (a mixture of Span 80 and Tween 80 with a mass ratio of 1:2) and 0.34 g of APS were added to 100 mL of distilled water and stirred homogeneously to form the aqueous phase. Second, the oil and aqueous phases were mixed together and stirred with a high-speed homogenizer (7000 rpm) for 10 min to obtain a homogeneous emulsion. Finally, the emulsion was transferred to a 250 mL three-neck flask equipped with a 300 rpm stirring device, and 20 mL of 0.26 g NaHSO_3_ solution was added dropwise to the above flask. The redox-initiated polymerization was carried out at 45 °C for 5 h. After polymerization, the product was filtered, washed, and dried to obtain the MPCM microcapsule.

#### 2.2.3. Preparation of MPCM/EG Phase Change Composites

An absorption polymerization technique was used to produce the MPCM/EG phase change composites, as shown in Scheme 1b. First, propyl palmitate and MMA monomer were mixed to form the oil phase and dropped into a certain amount of EG for sufficient absorption in no less than 30 min. Second, 1.5 g of emulsifier (a mixture of Span 80 and Tween 80 with a mass ratio of 1:2) and 0.34 g of APS were added to 100 mL of distilled water and stirred homogeneously to form the aqueous phase. Finally, the aqueous phases and EG, which had absorbed the oil phase, were transferred to a 250 mL three-neck flask equipped with a stirring device, and 20 mL of 0.26 g NaHSO_3_ solution was added dropwise to the above flask. The redox-initiated polymerization was carried out at 45 °C at a steady stirring speed of 300 rpm for 5 h. After polymerization, the products were filtered, washed, and dried to obtain the MPCM/EG phase change composites.

The resulting composites are designated as MPCM-x/EG-y, where x (x = 50, 70, and 80) is the theoretical propyl palmitate content in the oil phase (mixture of propyl palmitate and MMA monomer) and y (y = 6, 10, 15, and 25) is the theoretical EG content in the MPCM/EG composites. For example, MPCM-80/EG-6 shows that the sample contains 6 wt% of EG and 94 wt% of oil phase, with propyl palmitate making up 80 wt% of the oil phase (i.e., the theoretical loading of propyl palmitate in the composite is 75.2 wt%). The detailed formulations of the MPCM/EG composites are listed in Table 1.

For comparison, the MPCM@EG composites were prepared by the direct absorption of MPCM microcapsules by EG. The MPCM@EG composites were obtained by adding EG to the MPCM emulsion prepared in Section 2.2.2 and absorbing for 24 h.

### 2.3. Characterization

#### 2.3.1. Scanning Electron Microscopy (SEM)

Field emission scanning electron microscopy (FE-SEM, JSM-7401, JEOL Ltd., Tokyo, Japan) was used to study the microscopic morphologies of the composites. The surface of each sample was coated with gold before observation.

#### 2.3.2. Fourier-Transform Infrared Spectroscopy (FTIR)

A Nicolet 6700 Fourier-transform infrared (FTIR) spectrometer (Thermo Fisher Scientific, Waltham, MA, USA) was used to test the composition of the composites. The samples were mixed with KBr powder, then ground and pressed for testing. Each sample was scanned using 32 cumulative scans with a spectral resolution of 4 cm^−1^ and a wavenumber range of 4000–400 cm^−1^.

#### 2.3.3. Thermal Properties

The thermal properties of the composites were measured by a differential scanning calorimeter (DSC, NETZSCH Scientific Instruments Trading (Shanghai) Ltd., Shanghai, China). First, 2–3 mg samples were loaded into aluminium pans. The tests were performed in a flowing nitrogen atmosphere of 100 mL/min with a heating/cooling rate of 10 °C/min and a working temperature of −10–50 °C.

The encapsulation ratio (R) of the composites was evaluated using Equation (1):

(1) $$R=\frac{{\Delta H}_{m,Com}}{{\Delta H}_{m,propyl\;palmitate}}\times 100\%$$

where ΔH_m,Com_ was the obtained melting enthalpy of the composites, and ΔH_m,propyl palmitate_ was the obtained melting enthalpy of propyl palmitate.

The encapsulation efficiency (E) of the composites was evaluated using Equation (2):

(2) $$E=\frac{{\Delta H}_{m,Com}}{{\Delta H}_{m,propyl\;palmitate}{\cdot \varphi }_{Propyl\;palmitat}}\times 100\%=\frac{R}{\varphi_{Propyl\;palmitate}}\times 100\%$$

where R was derived from Equation (1), and φ_Propyl palmitate_ represented the theoretical loading of propyl palmitate in the composites.

#### 2.3.4. Thermogravimetric Analysis (TGA)

Thermogravimetric analysis (TGA) was carried out using a thermal analyzer (NETZSCH Scientific Instruments Trading (Shanghai) Ltd., Shanghai, China) to evaluate the thermal stability of the composites. The tests were performed in a nitrogen atmosphere (50 mL/min flow rate) with a heating rate of 10 °C/min from 35 to 700 °C.

#### 2.3.5. Thermal Conductivity Analysis

The thermal conductivity (λ, W/(m·K)) of the composites was determined using Equation (3):

(3) $$\lambda =\alpha \times \rho \times C_{p}$$

where α, ρ, and C_p_ are the measured thermal diffusion coefficient (m^2^/s), density (kg/m^3^), and specific heat capacity (J/(kg·K)) of the composites, respectively. The samples were cold-pressed into small flat cylinders with a diameter of 12.7 mm and a thickness of 2 mm. The mass and volume were recorded to obtain the density of the sample. α was measured by a laser flash thermal conductivity analyzer (LFA467, NETZSCH Scientific Instruments Trading (Shanghai) Ltd., Shanghai, China) with an accuracy of ±3%. C_p_ was determined by DSC. Each sample was measured three times, and the average was determined.

#### 2.3.6. Thermal Cycle Reliability

The thermal cycle reliability of the composites was evaluated by DSC cycling tests, which consisted of 50 heating/cooling cycles in the temperature range of −20–60 °C. To evaluate the thermal cycle reliability of the composites in the actual environment, an open environmental cycling test was also conducted: the composite was placed in a refrigerator at −20 °C for 30 min and then transferred to an oven at 60 °C for another 30 min. After 50 cycles, DSC was performed to characterize the thermal cycle stability of the sample.

## 3. Results

### 3.1. Morphological and Structural Analysis

**Figure 1:**
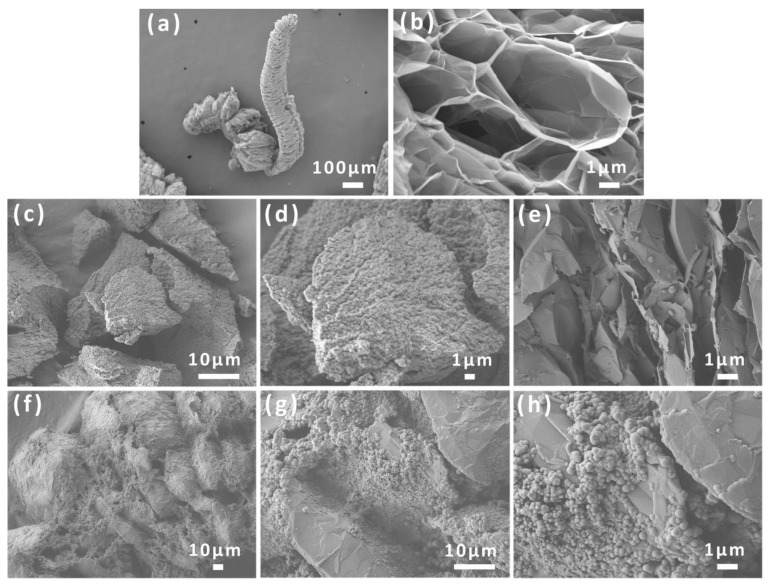
SEM images of (**a**,**b**) pure EG, (**c**,**d**) PMMA-microencapsulated MPCM microcapsules at different magnifications, (**e**) MPCM@EG composite, and (**f**–**h**) MPCM/EG composite at different magnifications.

SEM analysis was used to investigate the morphologies of MPCM microcapsules and MPCM/EG composites, as shown in Figure 1. Pure EG shows a porous, loose, and continuous “worm-like” network structure consisting of a large number of disordered graphite nanosheets interconnected by van der Waals interactions, and the average pore size is larger than 10 μm (Figure 1a,b). The SEM photos of the PMMA/propyl palmitate microcapsules (MPCMs) are shown in Figure 1c,d. The prepared MPCM microcapsules were well dispersed, with spherical and regular dense morphology, and the diameters of the microcapsules were mainly between 200 and 300 nm, indicating that PMMA successfully encapsulated propyl palmitate.

The combination pathway between EG and the MPCM microcapsules has an important influence on the morphology of the formed composites. There are two ways to introduce EG as an inorganic, thermally conductive skeleton into phase change composites. When the absorption polymerization method was employed (i.e., EG first absorbed the MMA monomer and propyl palmitate before initiating the polymerization reaction), the synthesized MPCM/EG composites retained the “worm-like” continuous structure of pure EG (Figure 1f), accompanied by the formation of MPCM microcapsules. Figure 1f–h show the morphologies of the MPCM/EG composites at different magnifications. The MPCM microcapsules were generated in situ and covered the entire porous structure of the EG networks during polymerization, with the EG surfaces also covered by a large number of microcapsules, indicating that the propyl palmitate was successfully encapsulated by the PMMA shell in the EG networks. The SEM results reveal that the expanded and well-developed porous structure of EG contributes to the high specific surface area and, thus, promotes the bulk absorption of the oil phase (a mixture of MMA and propyl palmitate) because of the capillary effect of the 3D EG networks, which further facilitates the in situ generation of microcapsules in the EG networks. The formed microcapsules are dispersed in the EG networks, and the dual encapsulation effect of the PMMA shell and the EG structure is essential for maintaining the enthalpy and preventing leakage during phase change.

A control experiment was performed for comparison. The MPCM microcapsules were first prepared to realize the encapsulation of PMMA on propyl palmitate, and then the MPCM@EG composites were formed by the direct absorption of MPCM microcapsules by EG, and the SEM result is shown in Figure 1e. The microcapsules are mainly attached to the exposed surface or in the gaps of the graphite sheets, which means that most of the microcapsules are not absorbed into the network skeleton of EG. Therefore, the direct absorption of MPCM microcapsules by EG is extremely weak and cannot effectively absorb the microcapsules in large quantities; thus, the MPCM@EG composite may result in a poor phase change effect.

Consequently, the absorption polymerization was proved to be a more suitable combination route than the direct absorption of MPCM microcapsules via EG, which facilitates the encapsulation of propyl palmitate and, thus, contributes to the thermal properties of the composites, as will be discussed subsequently.

**Figure 2:**
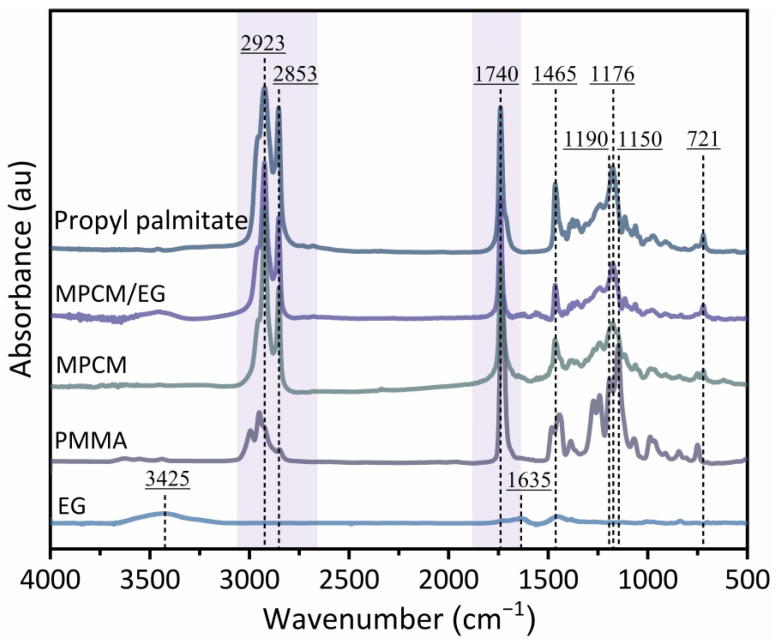
FTIR spectra of propyl palmitate, PMMA, EG, MPCM, and MPCM/EG.

To confirm the chemical composition, Figure 2 displays the FTIR spectra of MPCM and MPCM/EG composites. Figure 2 also includes the FTIR spectra of propyl palmitate, PMMA, and EG for comparison. The FTIR spectrum of propyl palmitate shows characteristic absorption peaks at 2923 and 2853 cm^−1^ that correspond to the asymmetric and symmetric stretching vibrations of the C–H bond, respectively; a peak at 1740 cm^−1^ that corresponds to the stretching vibration of the C=O carbonyl group; a peak at 1176 cm^−1^ that corresponds to the stretching vibration of the C–O bond; and peaks at 1465 and 721 cm^−1^ that correspond to the in-plane and out-of-plane bending vibrations of the C–H group, respectively. In the FTIR spectrum of PMMA, the peak at 1730 cm^−1^ corresponds to the stretching vibration of the C=O carbonyl group, and the peaks at 1190 and 1150 cm^−1^ correspond to the stretching vibration of the C–O ester group. The FTIR spectrum of pure EG exhibits no characteristic absorption peaks. The broad absorption peak at 3425 cm^−1^ and the narrow absorption peak at 1635 cm^−1^ are derived from water adsorbed in EG. The FTIR spectra of both MPCM and MPCM/EG composites show all the characteristic peaks of propyl palmitate and PMMA, and there is no shift in peak positions or generation of new characteristic absorption peaks. These results indicate the chemical composition of both MPCM and MPCM/EG composites, where propyl palmitate is physically encapsulated in the composite without chemical modification.

### 3.2. Formation Mechanism

The encapsulation mechanism of the MPCM/EG composites prepared by the absorption polymerization process is shown in Scheme 1: the oil phase formed by the MMA monomer and propyl palmitate is first absorbed into the 3D porous network structure of EG by capillary forces, and then the EG that absorbed the oil phase is distributed in the aqueous phase. The MMA monomer and propyl palmitate form micelle-encapsulated small oil droplets in the presence of an emulsifier. Due to different polarities, MMA with shorter chain segments is wrapped around the outer surface of the oil droplet, while propyl palmitate is in the center of the droplet. During polymerization, the water-soluble oxidation-reduction initiator APS-NaHSO_3_ in the aqueous phase diffuses to the water/oil interface of the EG layer and further diffuses to the surface of the oil droplets. Finally, the MMA monomers are polymerized to produce the microcapsules, with a PMMA shell encapsulating a propyl palmitate core in the EG networks. The expanded and 3D porous network structure of EG with large specific surface areas provides ample space for the formation of microcapsules. As a result, a large number of microcapsules are more easily formed that fill and cover the network structure of EG. The MPCM/EG composites prepared by the absorption polymerization retain the intact continuous “worm-like” structure of EG, while the MPCM microcapsules are densely dispersed in the 3D porous EG networks. However, the direct absorption of MPCM microcapsules by EG to prepare the MPCM@EG composites could not provide sufficient absorption capacity; therefore, the absorption effect is rather poor.

In summary, the preparation of the MPCM/EG composites by the absorption polymerization strategy is more efficient in promoting the formation of microcapsules in large quantities, with the entire porous structure of EG being covered by the formed MPCM microcapsules during polymerization. In the MPCM/EG composites, propyl palmitate as the PCM core helps to improve the thermal storage capacity; the doublet encapsulation structure of the PMMA shell and EG networks helps to prevent leakage; and EG helps to improve the thermal conductivity and maintain the shape stability of the composites. The newly developed absorption polymerization strategy is particularly promising for practical applications owing to its accessibility, high efficiency, and applicability to organic PCMs.

### 3.3. Thermal Properties

**Figure 3:**
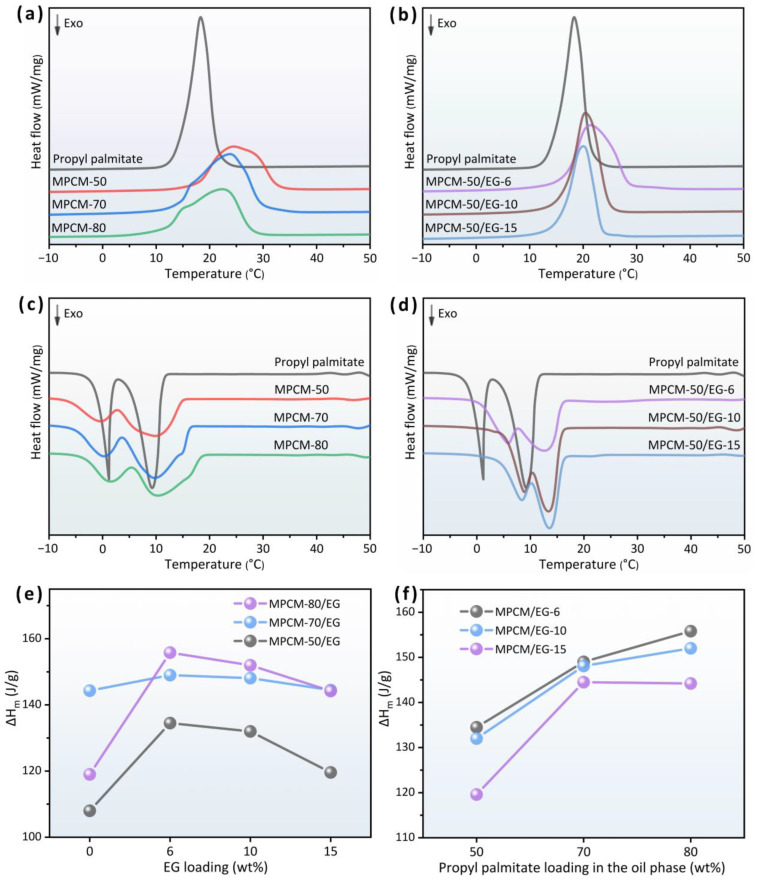
DSC (**a**,**b**) heating and (**c**,**d**) cooling curves of propyl palmitate, MPCM, and MPCM/EG composites; (**e**) melting enthalpy of MPCM/EG composites as a function of EG loading; (**f**) melting enthalpy of MPCM/EG composites as a function of propyl palmitate loading in the oil phase.

The thermal properties of propyl palmitate, MPCM, MPCM@EG, and MPCM/EG composites were evaluated by DSC, with corresponding curves of the heating and cooling processes shown in Figure 3. Table 2 summarizes the related phase change enthalpies, peak melting and solidification temperatures, theoretical loading of propyl palmitate in the composites (φ_Propyl palmitate_), encapsulation ratio (R), and encapsulation efficiency (E) of the composites.

As shown in Table 2, the average melting and freezing enthalpies of propyl palmitate were 168.3 and 165.7 J/g, respectively, and the corresponding peak melting and freezing temperatures were 18.3 and 9.2 °C, respectively. As shown in Figure 3a–d, the melting and freezing curves of the MPCM composites were similar to the phase change behavior of propyl palmitate, indicating that no chemical reaction occurred between propyl palmitate and the PMMA shell. The peak melting and solidification temperatures of the MPCM composites were both higher than those of propyl palmitate, which may be attributed to the thermal resistance of the PMMA shell. The enthalpies of the MPCM composites first increased and then decreased as the amount of propyl palmitate in the MMA/propyl palmitate mixture increased from 50 wt% to 80 wt%. The maximum latent heat of the MPCM composites was 144.3 J/g when the propyl palmitate content was 70 wt%. Compared with the MPCM composites, the peak melting temperatures decreased and the peak freezing temperatures increased with the increase in the EG content in the MPCM/EG composites, which indicated that the introduction of EG in the MPCM/EG composites helped to enhance the heat transfer, so as to improve the thermal conduction of the MPCM/EG composites. When the content of EG in the MPCM/EG composites was kept constant, the change of the propyl palmitate content in the composites had little influence on the phase transition temperatures of the MPCM/EG composites (Table 2).

As shown in Figure 3e and Table 2, the enthalpies of the MPCM/EG composites increased when the content of propyl palmitate in the MMA/propyl palmitate mixture increased from 50 wt% to 80 wt%, which was mainly due to the increase in the absorption of propyl palmitate by unit EG in the oil phase, thus contributing to the increase in the enthalpies of the MPCM/EG composites. Correspondingly, the overall enthalpies of the MPCM/EG composites gradually decreased when the content of EG increased from 6 wt% to 15 wt%, which was mainly due to the competition of excessive EG during oil phase absorption, resulting in a reduction of the oil phase absorbed by unit EG. This, in turn, leads to a decrease in the enthalpies of the MPCM/EG composites after polymerization. What is noteworthy is that the enthalpies and encapsulation rates of the MPCM/EG composites were much higher than those of the corresponding MPCM composites, which reveals that the absorption polymerization through EG enhances the encapsulation of propyl palmitate by PMMA. However, in contrast, the enthalpy of MPCM-50@EG-6 by direct absorption of MPCM microcapsules through EG was only 24.1 J/g (Table 2).

The encapsulation rate (R) of the MPCM/EG composites was evaluated by equation (1), and the results are listed in Table 2. The encapsulation rate of the MPCM/EG composites increased with the content of propyl palmitate in the MMA/propyl palmitate mixture. Both MPCM-80/EG-6 and MPCM-80/EG-10 have enthalpies exceeding 150 J/g and encapsulation rates exceeding 90% when the propyl palmitate content reaches 80 wt%. The encapsulation efficiency (E) of the MPCM/EG composites evaluated by Equation (2) decreases dramatically with the increase in propyl palmitate content, but they are all higher than 100%. This indicates that the content of MMA is more than needed for the encapsulation of propyl palmitate; therefore, the enthalpy of the MPCM/EG composites is not in proportion to the theoretical loading of propyl palmitate (φ_Propyl palmitate_). On the one hand, MMA is prone to volatilization and cause losses; on the other hand, it may also homopolymerize into PMMA, which leads to an encapsulation rate of MPCM/EG higher than the theoretical loading of propyl palmitate and ultimately to an encapsulation efficiency higher than 100%.

As shown in Figure 3f, the entire enthalpy increment of MPCM/EG-6, -10, and -15 composites increased rapidly as the propyl palmitate content increased from 50 to 70 wt% and slowed down as the propyl palmitate content increased from 70 to 80 wt%. In particular, the enthalpy no longer increases when the EG content is at 15 wt% (the enthalpies of MPCM-70/EG-15 and MPCM-80/EG-15 are 144.5 and 144.2 J/g, respectively). This indicates that the lower MMA content is not sufficient to form a complete PMMA shell, and further increasing the content of propyl palmitate in the MMA/propyl palmitate mixture is of little significance. Therefore, the optimal content of propyl palmitate should be 80 wt%. To sum up, the MPCM/EG composite with the highest enthalpy in our study is MPCM-80/EG-6, with a highest enthalpy of 155.8 J/g and an encapsulation rate of 92.6%, which can maintain sufficient phase transition and thermal storage capacity.

### 3.4. TGA Analysis

**Figure 4:**
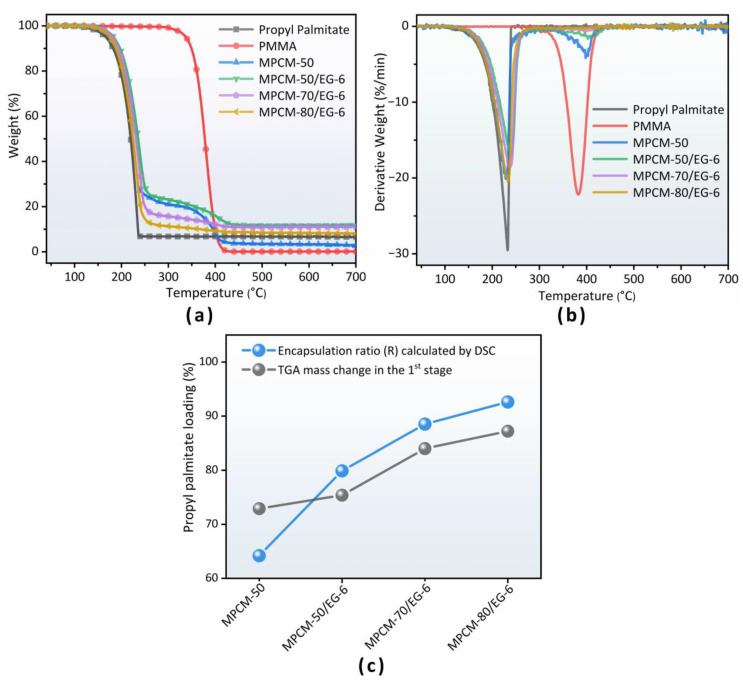
(**a**,**b**) TGA and the corresponding DTG curves of propyl palmitate, PMMA, MPCM, and MPCM/EG composites in a nitrogen atmosphere; (**c**) Comparison of the encapsulation ratio (R) calculated by DSC and the TGA mass change in the 1st stage.

The thermal stability of propyl palmitate, PMMA, MPCM, and MPCM/EG composites was evaluated by TGA, as shown in Figure 4. The temperature at 5 wt% mass loss (T_5%_), the maximum mass loss rate temperatures (T_max1_ and T_max2_), the mass change before 250 °C (1st stage), and the residual weight at 700 °C are given in Table 3.

As shown in Figure 4 and Table 3, the weight loss process of propyl palmitate showed a single weight-loss stage with a T_5%_ of 167.0 °C and a T_max_ of 232.6 °C. The mass change before 250 °C is about 93.2%, which corresponds to the volatilization of propyl palmitate. The residual weight of propyl palmitate at 700 °C is about 6.8%, which is mainly derived from industrial impurities. PMMA also undergoes a single-step degradation process with a higher T_5%_ of 337.5 °C and a T_max_ of 382.3 °C, leaving almost no residue at 700 °C.

The MPCM and MPCM/EG composites showed similar thermal degradation behavior, with two weight loss stages observed in both of their TGA curves (Figure 4a,b), which were attributed to the volatilization of propyl palmitate and the decomposition of the PMMA shell, respectively. The T_5%_ and T_max2_ of MPCM-50 were 6.4 and 14.3 °C higher than those of propyl palmitate and PMMA, respectively, while its T_max1_ was slightly lower, indicating that the presence of the PMMA shell improved the thermal stability of MPCM-50 to some extent. Notably, the T_5%_, T_max1_, and T_max2_ of the MPCM-50/EG-6 composite are 183.3, 239.5, and 403.3 °C, respectively, which are 9.9, 9.7, and 6.7 °C higher, respectively, than those of MPCM-50 and 16.3, 6.9, and 21.0 °C higher, respectively, than those of propyl palmitate and PMMA. Evidently, the introduction of EG into MPCM/EG by absorption polymerization significantly improved the thermal stability of the composites. Furthermore, the mass change of the MPCM/EG composites in the first stage of TGA agrees well with the encapsulation ratio (R) calculated by DSC, as shown in Figure 4c, which is evidence of the successful preparation and encapsulation effects of the MPCM/EG composites, along with the FTIR and SEM results.

At 700 °C, the residual fractions of the MPCM/EG composites were higher than the theoretical content of EG (6 wt%), indicating that the effect of the physical barriers of the EG layers and their interaction with the PMMA shell enhanced the carbonization of PMMA, resulting in more char.

### 3.5. Thermal Conductivity Enhancement

**Figure 5:**
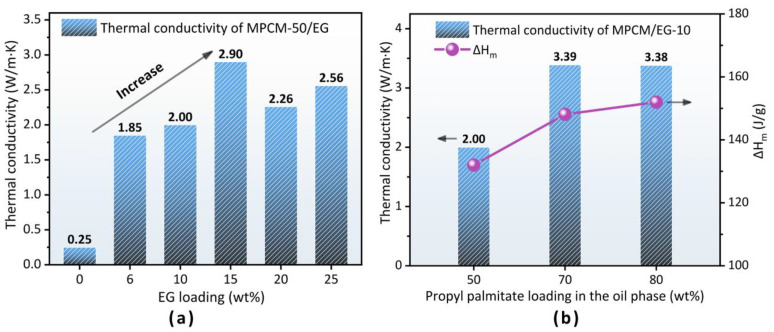
(**a**) Thermal conductivity of the MPCM-50/EG composites as a function of EG loading; (**b**) Thermal conductivity and enthalpy of the MPCM/EG-10 composites as a function of propyl palmitate loading in the oil phase.

Thermal conductivity is a key factor in assessing the efficiency of heat transfer between the heat source and its surrounding environment. Figure 5a displays the thermal conductivity of MPCM-50/EG composites as a function of EG loading. Propyl palmitate has an initial low thermal conductivity of 0.20 W/(m·K). The thermal conductivity of MPCM-50 microcapsules is not much better than that of propyl palmitate, which is only 0.25 W/(m·K), indicating that the PMMA shell has limited effect on improving the thermal conductivity of the MPCM composites. In comparison, after combination with the net-like porous structure of EG, all the MPCM-50/EG composites exhibit a substantial increase in thermal conductivity of more than 1.50 W/(m·K), which is much higher than that of propyl palmitate and MPCM-50. Thus, the thermal conductivity of the MPCM/EG composites was dramatically enhanced.

The thermal conductivity of the MPCM-50/EG composites increased dramatically with the increase of EG loading when the EG content was below 15%. A maximum thermal conductivity of 2.90 W/(m·K) was achieved at 15% EG loading, which is about 14.5 times higher than that of propyl palmitate. However, the thermal conductivity of the composites began to decrease when the loading of EG was further increased. These results suggest that the interconnected long-chain network structure of the introduced EG, which is well preserved, facilitates the formation of the thermally conductive pathways in the composites when the EG loading is below 15%. Nevertheless, further increases of EG will bring numerous unfilled air bubbles into the composite, thereby forming many separate closed spaces, which prevents further improvement in the thermal conductivity of the composites. In addition, as shown in the previous DSC results, the enthalpy and encapsulation ratio of the composites decrease rapidly when EG exceeds 10%. Therefore, the amount of EG in the MPCM/EG composites should be controlled to 10% or less to ensure high enthalpy while maintaining high thermal conductivity.

Figure 5b shows the thermal conductivity and enthalpy of the MPCM/EG-10 composites as a function of propyl palmitate loading. When the loading of propyl palmitate was increased from 50% to 80%, the thermal conductivity of the MPCM-50/EG-10, MPCM-70/EG-10, and MPCM-80/EG-10 composites increased significantly to 2.00, 3.39, and 3.38 W/(m·K), respectively, which were 10, 17, and 16 times higher, respectively, than that of propyl palmitate. The thermal conductivity of the composites remains essentially constant when the loading of propyl palmitate is kept at 70% or 80%, while the composites have a maximum enthalpy of 152.0 J/g when the loading of propyl palmitate is 80%. Taking into account the latent heat and encapsulation reliability, MPCM-80/EG-10 has the optimal combined performance: a thermal conductivity of 3.38 W/(m·K), a phase change enthalpy of 152.0 J/g, and an encapsulation ratio of 90.3%, which was applied in subsequent tests.

### 3.6. Leakage Tests

**Figure 6:**
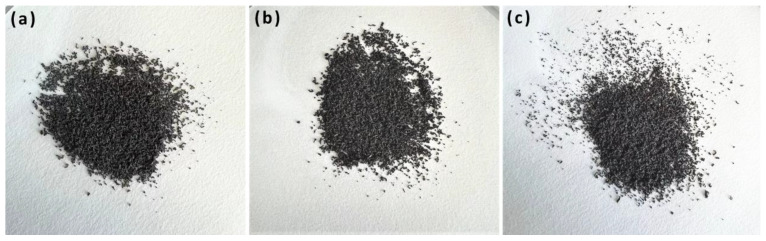
Digital photos of MPCM-80/EG-10 heated on a hotplate at (**a**) room temperature (~25 °C), (**b**) 60 °C for 30 min, and (**c**) 80 °C for 30 min.

Leakage tests were used to determine the structural stability of the MPCM-80/EG-10 composite. Propyl palmitate, which has a melting point of ~18.3 °C, is in liquid form at room temperature (~25 °C), while the MPCM-80/EG-10 composite maintains a powdery solid form. Before leakage tests, MPCM-80/EG-10 was sufficiently refrigerated at −20 °C to complete the solidification process, then quickly transferred to a clean hot plate and heated at 60 and 80 °C, separately, for at least 30 min to ensure a sufficient melting process. The test temperatures were above the melting point of propyl palmitate, but did not lead to the decomposition of PMMA. As shown in Figure 6, MPCM-80/EG-10 retained its initial powdery form at both 60 and 80 °C without shape change or leakage of propyl palmitate. The results demonstrate that the dual encapsulation of the PMMA shell and the network structure of EG provide adequate mechanical strength to prevent leakage and withstand the volumetric change of propyl palmitate during phase transition from solid to liquid, thereby achieving superior shape stability.

### 3.7. Thermal Cycle Reliability

The thermal cycle reliability of phase change composites is an important criterion for evaluating the encapsulation stability and longevity. The foregoing results show that the MPCM/EG composites fabricated in this study exhibit both high enthalpy and high thermal conductivity. Correspondingly, the thermal cycle reliability of the MPCM/EG composites is also noted.

**Figure 7:**
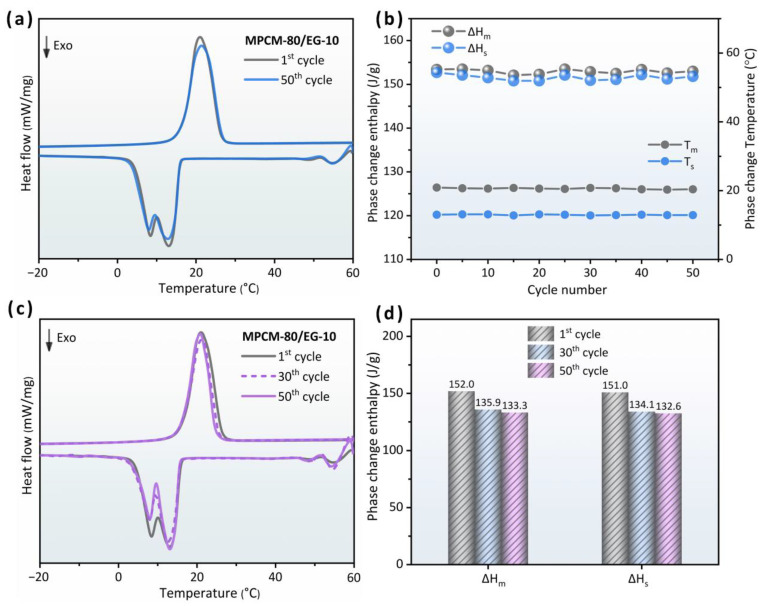
(**a**) Thermal cycle reliability and (**b**) cycle performance (including the parameters of phase change enthalpy and phase change temperature) of MPCM-80/EG-10 before and after 50 DSC heating/cooling cycles; (**c**) DSC curves and (**d**) enthalpy evolution of MPCM-80/EG-10 before and after 50 cycles under oven/refrigerator conditions.

First, the thermal cycle reliability of the MPCM-80/EG-10 composite was evaluated by DSC with 50 heating/cooling cycles in the temperature range of −20–60 °C. Since the DSC measurement has high testing accuracy, it can control the consistency of the environment and reduce the errors caused by environmental changes and experimental operations on the results, thereby allowing a better evaluation of the thermal cycle reliability. As shown in Figure 7a,b, the heating/cooling curves of MPCM-80/EG-10 are generally similar to those before cycling. Thus, the MPCM-80/EG-10 composite showed steady phase change enthalpies over 50 DSC heating/cooling cycles. In addition, other important parameters of the MPCM-80/EG-10 composite, including ΔH_s_, T_m_, and T_s_, remained almost unchanged following 50 DSC heating/cooling cycles, further verifying the excellent cycling characteristics of the composites.

In addition, an open environmental cycling test was performed to evaluate the thermal cycle reliability of the composites in an actual environment by subjecting them to open oven/refrigerator conditions: the composite is placed in a −20 °C refrigerator for 30 min to fully complete the solidification process, then moved to a 60 °C oven for 30 min to fully complete the melting process. Any potential PCM leakage during cycling was absorbed with filter paper at the bottom to simulate losses in the actual application environment. After 50 cycles, DSC was conducted to characterize the thermal cycle stability of the sample. The DSC curves of MPCM-80/EG-10 prior to and after 50 cycles in the oven/refrigerator are shown in Figure 7c, with detailed data displayed in Figure 7d.

As shown in Figure S1, the MPCM-80/EG-10 composite was in powder form before cycling, while adhesion occurred among some particles during cycling. Because of the volume change caused by the phase transition of PCM, a small amount of liquid propyl palmitate in the molten state may overflow onto the surface of MPCM-80/EG-10, causing the adhesion of nearby particles. Nevertheless, the adhesion was slight, and there was no obvious leakage of PCM from the filter paper surface, indicating that the MPCM-80/EG-10 composite was able to maintain shape stability during extended cycling. As shown in Figure 7c,d, the initial enthalpy of MPCM-80/EG-10 before cycling is 152.0 J/g. The enthalpy after the 30th–50th DSC cycles is basically stable above 130.0 J/g, which can meet the requirements of application in practical situations. Furthermore, the primary peaks of MPCM-80/EG-10 in the FTIR spectra remained unchanged after 50 heating/cooling cycles (Figure S2). The enthalpy retention capability of the prepared MPCM/EG composites is not much influenced by the heating/cooling cycle frequency, demonstrating that the composites, by absorption polymerization, simultaneously achieve high enthalpy, high thermal conductivity, low leakage, and good cycle reliability in long-term applications.

## 4. Conclusions

In this study, we propose a simple and efficient absorption polymerization strategy for preparing MPCM/EG composites with high thermal conductivity, high enthalpy, excellent thermal stability, low leakage, and good thermal cycle reliability, which was achieved by a two-step procedure: (1) absorption of MMA and propyl palmitate by EG and (2) polymerization of MMA to encapsulate propyl palmitate to form the MPCM microcapsules with core-shell structures in the 3D EG networks. The SEM and FTIR results demonstrated the formation of the PMMA-microencapsulated propyl palmitate microcapsules in the EG networks, which solved the leakage problem of the PCM core, while enhancing the thermal conductivity and imparting good thermal properties and cycle reliability to the composites. The MPCM/EG composites exhibited excellent overall performance, including a thermal conductivity of 3.38 W/(m·K), latent heat of up to 152.0 J/g (encapsulation ratio of 90.3%), outstanding thermal stability, and long-term thermal cyclic reliability at EG loading of 10% and propyl palmitate of 80%. This work provides new insights into the design, fabrication, and enhancement strategies for highly integrated phase change composites with prospective applications in solar energy conservation, smart buildings, waste heat treatment, and thermal protection of microelectronic devices.

## Figures and Tables

**Table 1:** The formulation of the MPCM/EG phase change composites.

Sample	mPCM/(mPCM + mMMA) (wt%)	EG (wt%)
MPCM-50	50	—
MPCM-70	70	—
MPCM-80	80	—
MPCM-50/EG-6	50	6
MPCM-70/EG-6	70	6
MPCM-80/EG-6	80	6
MPCM-50/EG-10	50	10
MPCM-70/EG-10	70	10
MPCM-80/EG-10	80	10
MPCM-50/EG-15	50	15
MPCM-70/EG-15	70	15
MPCM-80/EG-15	80	15
MPCM-50/EG-20	50	20
MPCM-50/EG-25	50	25
MPCM-50@EG-6	50	6

**Table 2:** DSC results of propyl palmitate, MPCM, MPCM@EG, and MPCM/EG composites under nitrogen atmosphere.

Sample	ΔH_m_ (J/g)	ΔH_s_ (J/g)	T_m_ (°C)	T_s_ (°C)	φ_Propyl palmitate_^1^ (%)	Encapsulation Ratio ^2^ (%)	Encapsulation Efficiency ^3^ (%)
Propyl palmitate	168.3	165.7	18.3	9.2	—	—	—
MPCM-50	108.0	106.2	24.5	9.8	50.0	64.2	128.3
MPCM-70	144.3	141.0	23.8	9.8	70.0	85.7	122.5
MPCM-80	119.0	118.3	22.4	10.3	80.0	70.7	88.4
MPCM-50/EG-6	134.5	133.1	21.4	12.6	47.0	79.9	170.0
MPCM-70/EG-6	149.0	145.0	21.5	12.4	65.8	88.5	134.5
MPCM-80/EG-6	155.8	154.6	21.4	12.3	75.2	92.6	123.1
MPCM-50/EG-10	132.0	133.8	20.4	13.4	45.0	78.4	174.3
MPCM-70/EG-10	148.1	146.1	20.7	13.2	63.0	88.0	139.7
MPCM-80/EG-10	152.0	151.0	20.8	13.0	72.0	90.3	125.4
MPCM-50/EG-15	119.6	118.2	20.0	13.5	42.5	71.1	167.2
MPCM-70/EG-15	144.5	142.9	20.4	13.5	59.5	85.9	144.3
MPCM-80/EG-15	144.2	143.5	20.3	13.3	68.0	85.7	126.0
MPCM-50@EG-6	24.1	22.2	18.0	14.5	47.0	14.3	30.5

^1^: φ_Propyl palmitate_ is the theoretical loading of propyl palmitate in the composites. ^2^: The encapsulation ratio (R) of the composites is evaluated by Equation (1). ^3^: The encapsulation efficiency (E) of the composites is evaluated by Equation (2).

**Table 3:** TGA data of propyl palmitate, PMMA, MPCM, and MPCM/EG composites.

Sample	T_5%_ °C	T_max1_ °C	T_max2_ °C	Mass Change (35–250 °C) (%)	Residue at 700 °C (%)
Propyl palmitate	167.0	232.6	—	93.2	6.8
PMMA	337.5	—	382.3	0.3	0.2
MPCM-50	173.4	229.8	396.6	72.9	3.5
MPCM-50/EG-6	183.3	239.5	403.3	75.4	11.6
MPCM-70/EG-6	180.4	237.5	396.0	84.0	9.4
MPCM-80/EG-6	176.7	233.3	395.8	87.2	8.1

## Data Availability

Not applicable.

## Supplementary Materials

The following supporting information can be downloaded at: https://www.mdpi.com/article/10.3390/polym15153192/s1, Figure S1: Digital images of the MPCM-80/EG-10 composite: (a) before cycling, and (b) after 50 cycles in the oven/refrigerator. Figure S2: FTIR spectra of the MPCM-80/EG-10 composite before/after 50 cycles in the oven/refrigerator.
