# Supplementary material for: Preparation and Thermal Properties of Propyl Palmitate-Based Phase Change Composites with Enhanced Thermal Conductivity for Thermal Energy Storage
Source: Polymers (Basel). 2023 Jul 27;15(15):3192. doi: 10.3390/polym15153192 (PMC10421114; doi:10.3390/polym15153192)
Supplement: Supplementary file 1 [file polymers-15-03192-s001.zip › polymers-2520706-SI.pdf]

Preparation and thermal properties of propyl palmitate-based phase change composites with enhanced thermal conductivity for thermal energy storage

Linzhi Yin, Min Zhao*, Rui Yang

Department of Chemical Engineering, Tsinghua University, Beijing 100084, PR China

* Correspondence to: Min Zhao (minzhaochn@126.com)

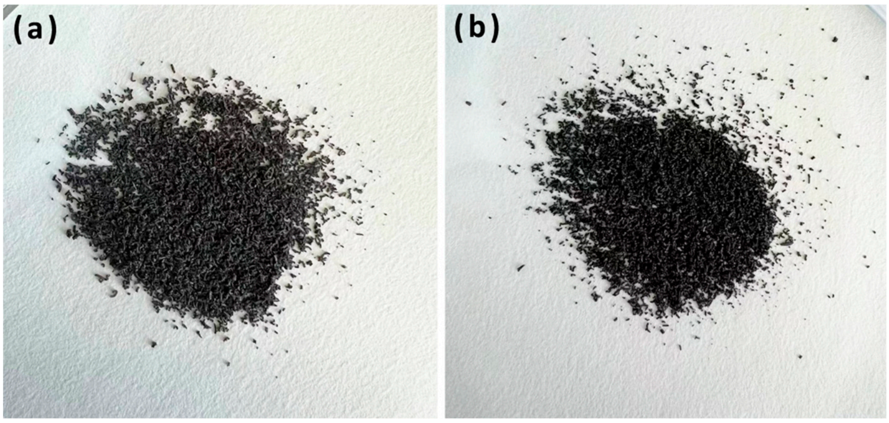

Figure S1. Digital images of the MPCM-80/EG-10 composite: (a) before cycling, and (b) after 50 cycles in the oven/refrigerator.

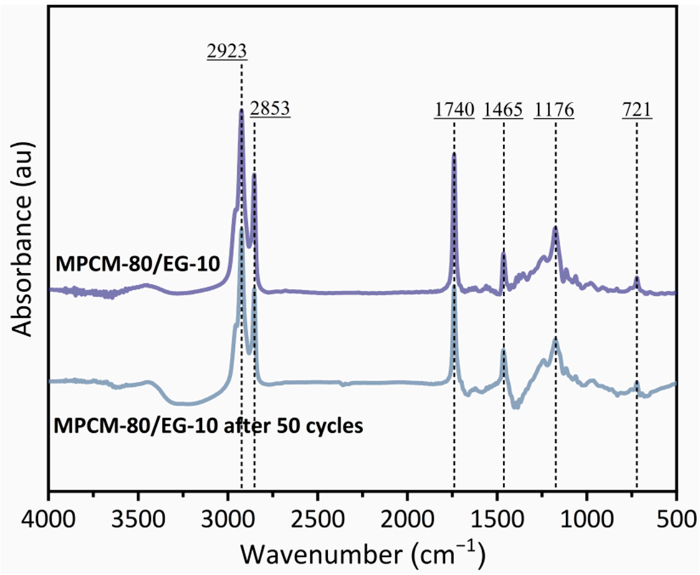

Figure S2. FTIR spectra of the MPCM-80/EG-10 composite before/after 50 cycles in the oven/refrigerator.
